# Supplementary material for: Time-series transcriptome analysis identified differentially expressed genes in broiler chicken infected with mixed Eimeria species
Source: Front Genet. 2022 Aug 8;13:886781. doi: 10.3389/fgene.2022.886781 (PMC9393255; doi:10.3389/fgene.2022.886781)
Supplement: Supplementary file 2 [file DataSheet1.ZIP › 7dpi_GO.Gsea.1625106495923/GOMF_PROTEIN_TYROSINE_KINASE_BINDING.html]

Details for gene set GOMF\_PROTEIN\_TYROSINE\_KINASE\_BINDING[GSEA]

|  || Dataset | TMM\_7dpi\_gct\_format\_7dpi\_gct\_format.Class\_4dpi.cls #PC\_versus\_NC.Class\_4dpi.cls #PC\_versus\_NC\_repos |
| Phenotype | Class\_4dpi.cls#PC\_versus\_NC\_repos |
| Upregulated in class | 0 |
| GeneSet | GOMF\_PROTEIN\_TYROSINE\_KINASE\_BINDING |
| Enrichment Score (ES) | -0.53925794 |
| Normalized Enrichment Score (NES) | -1.8684932 |
| Nominal p-value | 0.0 |
| FDR q-value | 0.09206645 |
| FWER p-Value | 0.6268 |
Table: GSEA Results Summary

  

Fig 1: Enrichment plot: GOMF\_PROTEIN\_TYROSINE\_KINASE\_BINDING      
 Profile of the Running ES Score & Positions of GeneSet Members on the Rank Ordered List

  

| SYMBOL | TITLE | RANK IN GENE LIST | RANK METRIC SCORE | RUNNING ES | CORE ENRICHMENT || 1 | SQSTM1 | na | 594 | 0.688 | -0.0326 | No |
| 2 | DUSP3 | na | 1155 | 0.470 | -0.0678 | No |
| 3 | SIRPA | na | 1725 | 0.326 | -0.1074 | No |
| 4 | CD4 | na | 2019 | 0.268 | -0.1252 | No |
| 5 | SHC4 | na | 2866 | 0.143 | -0.1925 | No |
| 6 | ACVR1 | na | 3013 | 0.125 | -0.2016 | No |
| 7 | RACK1 | na | 3023 | 0.123 | -0.1993 | No |
| 8 | DAZAP2 | na | 3063 | 0.118 | -0.1996 | No |
| 9 | HYAL2 | na | 3478 | 0.065 | -0.2326 | No |
| 10 | ZPR1 | na | 3504 | 0.063 | -0.2332 | No |
| 11 | PRR7 | na | 3538 | 0.060 | -0.2345 | No |
| 12 | ARHGEF16 | na | 3563 | 0.057 | -0.2351 | No |
| 13 | EPHA4 | na | 3696 | 0.041 | -0.2451 | No |
| 14 | GAS6 | na | 3932 | 0.012 | -0.2645 | No |
| 15 | MYOC | na | 4194 | -0.016 | -0.2859 | No |
| 16 | RNF41 | na | 4213 | -0.019 | -0.2870 | No |
| 17 | NRTN | na | 4562 | -0.057 | -0.3147 | No |
| 18 | PCNA | na | 5081 | -0.111 | -0.3553 | No |
| 19 | CPNE3 | na | 5102 | -0.113 | -0.3542 | No |
| 20 | YWHAG | na | 5436 | -0.142 | -0.3785 | No |
| 21 | ANGPT1 | na | 5440 | -0.142 | -0.3752 | No |
| 22 | SHC2 | na | 5501 | -0.149 | -0.3766 | No |
| 23 | ELMO2 | na | 5539 | -0.153 | -0.3759 | No |
| 24 | KHDRBS1 | na | 5694 | -0.169 | -0.3845 | No |
| 25 | SHC1 | na | 5796 | -0.179 | -0.3885 | No |
| 26 | NRG1 | na | 5983 | -0.200 | -0.3992 | No |
| 27 | PLCG1 | na | 6241 | -0.228 | -0.4150 | No |
| 28 | CDH5 | na | 6834 | -0.290 | -0.4573 | No |
| 29 | SOCS5 | na | 6883 | -0.295 | -0.4540 | No |
| 30 | CRK | na | 6900 | -0.297 | -0.4480 | No |
| 31 | DOK2 | na | 7091 | -0.319 | -0.4560 | No |
| 32 | ITGB1 | na | 7209 | -0.331 | -0.4575 | No |
| 33 | TREM2 | na | 7695 | -0.383 | -0.4886 | No |
| 34 | MAP3K7 | na | 7867 | -0.403 | -0.4929 | No |
| 35 | TJP2 | na | 8188 | -0.444 | -0.5086 | No |
| 36 | ANGPT4 | na | 8206 | -0.447 | -0.4989 | No |
| 37 | STAP1 | na | 8317 | -0.461 | -0.4967 | No |
| 38 | PTPN2 | na | 8320 | -0.461 | -0.4854 | No |
| 39 | NCK1 | na | 8321 | -0.461 | -0.4739 | No |
| 40 | FNTA | na | 8552 | -0.492 | -0.4809 | No |
| 41 | PTEN | na | 8620 | -0.503 | -0.4740 | No |
| 42 | ANGPT2 | na | 8861 | -0.532 | -0.4809 | No |
| 43 | TRAT1 | na | 9372 | -0.610 | -0.5084 | No |
| 44 | PIK3R2 | na | 9597 | -0.645 | -0.5111 | No |
| 45 | EIF3A | na | 9642 | -0.652 | -0.4986 | No |
| 46 | PTPN14 | na | 9688 | -0.659 | -0.4859 | No |
| 47 | PITPNM3 | na | 10000 | -0.712 | -0.4943 | No |
| 48 | HSP90AA1 | na | 10002 | -0.712 | -0.4767 | No |
| 49 | PTPN11 | na | 10560 | -0.822 | -0.5028 | No |
| 50 | CBLB | na | 10996 | -0.935 | -0.5160 | Yes |
| 51 | DOCK4 | na | 11087 | -0.960 | -0.4996 | Yes |
| 52 | PIK3R1 | na | 11265 | -1.023 | -0.4890 | Yes |
| 53 | NEDD9 | na | 11283 | -1.030 | -0.4648 | Yes |
| 54 | TRADD | na | 11354 | -1.059 | -0.4443 | Yes |
| 55 | CD247 | na | 11411 | -1.084 | -0.4220 | Yes |
| 56 | PTPN1 | na | 11465 | -1.103 | -0.3990 | Yes |
| 57 | SH2B3 | na | 11614 | -1.181 | -0.3820 | Yes |
| 58 | BANK1 | na | 11665 | -1.213 | -0.3560 | Yes |
| 59 | BMPR2 | na | 11698 | -1.238 | -0.3279 | Yes |
| 60 | FRS2 | na | 11717 | -1.256 | -0.2981 | Yes |
| 61 | CD2 | na | 11718 | -1.259 | -0.2668 | Yes |
| 62 | MST1 | na | 11743 | -1.281 | -0.2369 | Yes |
| 63 | TIAM1 | na | 11867 | -1.437 | -0.2115 | Yes |
| 64 | CBL | na | 11873 | -1.446 | -0.1759 | Yes |
| 65 | LRP4 | na | 11922 | -1.569 | -0.1409 | Yes |
| 66 | PITPNM2 | na | 11968 | -1.754 | -0.1010 | Yes |
| 67 | NOX4 | na | 11976 | -1.831 | -0.0560 | Yes |
| 68 | GAB2 | na | 12006 | -2.362 | 0.0003 | Yes |
Table: GSEA details [plain text format]

  

Fig 2: GOMF\_PROTEIN\_TYROSINE\_KINASE\_BINDING      
 Blue-Pink O' Gram in the Space of the Analyzed GeneSet

  

Fig 3: GOMF\_PROTEIN\_TYROSINE\_KINASE\_BINDING: Random ES distribution      
 Gene set null distribution of ES for **GOMF\_PROTEIN\_TYROSINE\_KINASE\_BINDING**
